# Synopsis of the Course of Lectures on Materia Medica and Pharmacy

**Published:** 1863-12

**Authors:** 


For sale by W. B. Keen & Co., 148 Lake Street, Chicago.

---

Synopsis of the Course of Lectures on Materia Medica and Pharmacy, Delivered in the University of Pennsylvania; with Three Lectures on the Modus Operandi of Medicines. By Joseph Carson, M.D. Third Edition Revised. Philadelphia: Blanchard & Lea. 1863.

This is an octavo volume of 244 pages, containing a simple outline or rather skeleton of the course of lectures given by Dr. Carson in the University of Pennsylvania. It is specially designed for the use of students attending that school; and certainly the greater part of its contents can possess very little interest or value to any one else. The only part of the work of real interest to the general practitioner, is the last 38 pages, comprising three lectures on "the Operation of Medicines through the medium of the Nervous System, and by Absorption." These three lectures are published in full, and contain a very interesting summary of facts and opinions in relation to the highly important subject under consideration.

